# Proteomic Investigation of Human Dental Pulp to Identify Individuals Who Are Pregnant

**DOI:** 10.1002/prca.70011

**Published:** 2025-05-31

**Authors:** Takumi Tsutaya, Kana Fujimoto, Yusuke Nakai, Naana Mori, Ran Iguchi, Akinori Moroi, Kunio Yoshizawa, Koichiro Ueki, Yayoi Kimura, Noboru Adachi

**Affiliations:** ^1^ Research Center for Integrative Evolutionary Science Graduate University for Advanced Studies (SOKENDAI) Hayama Kanagawa Japan; ^2^ Biogeochemistry Research Center Research Institute for Marine Resources Utilization Japan Agency for Marine‐Earth Science and Technology Yokosuka Kanagawa Japan; ^3^ Department of Oral and Maxillofacial Surgery Faculty of Medicine University of Yamanashi Chuo Yamanashi Japan; ^4^ Advanced Medical Research Center Yokohama City University Kanazawa, Yokohama Kanagawa Japan; ^5^ Department of Advancing Clinical Research Graduate School of Medicine University of Yamanashi Chuo Yamanashi Japan; ^6^ Department of Legal Medicine Interdisciplinary Graduate School of Medicine and Engineering University of Yamanashi Chuo Yamanashi Japan

**Keywords:** blood circulation, dental pulp, forensic identification, postpartum, pregnancy

## Abstract

Biomolecules preserved in dental pulp are increasingly being used to identify individuals in the context of forensics and archaeology. Despite the vast amount of research into host and pathogen DNA, the potential use of physiologically informative proteins preserved in dental pulp has rarely been studied. Here, we hypothesized that pregnancy‐specific proteins circulating in the blood could be identified from the dental pulp of postpartum individuals and this was investigated using eight human third molars extracted from four postpartum and three control individuals during clinical treatment. A total of 885 proteins were identified from these eight dental pulp samples using liquid chromatography coupled tandem mass spectrometry, whose gene ontology compositions were similar to previous studies. However, despite our hypothesis, pregnancy‐specific proteins were not identified from the dental pulp of postpartum individuals (*n* = 5, 4–12 months postpartum). Although the dental pulp proteomes obtained from three individuals postpartum ≤6 months were distinct from those of other individuals by principal component analysis (PCA), their driving proteins were less evident. Although our hypothesis was not supported, sample collection, protein extraction, and mass spectrometry analysis could be improved to explore the forensic application of detecting pregnancy‐specific proteins in dental pulp.

## 1 Introduction

The dental pulp is a reservoir of endogenous biomolecules that can be used in forensic science, such as for individual identification [1, 2, 3]. The dental pulp is the inner mesenchymal tooth tissue. Blood vessels and nerve fibers enter the pulp chamber via the root apex [4, 5]. Therefore, blood‐borne pathogens and host biomolecules can enter the pulp chamber and be well preserved within the pulp even after the death of the host individual and for a postmortem period [6]. In addition, the dental pulp is a semi‐closed system with only a tiny pore at the root apex, which prevents the degradation of endogenous biomolecules and contamination of exogenous biomolecules after the death of the host individual. Teeth are highly mineralized and mainly composed of enamel, which is the hardest tissue in the human body, and dentin. These are typical materials that are the most resistant to various postmortem degradation processes, such as decomposition and cremation. Therefore, the availability of teeth is generally higher than any other human tissue at crime scenes and archaeological settings. Analyses of endogenous host and pathogen DNA trapped in the dental pulp can be used to genotype and identify human bodies, determine sex, and detect bacterial infection both in the context of forensic and archaeological sciences [1, 2, 3,6, 7, 8].

**Summary**

- To investigate pregnancy‐specific proteins could be identified from the dental pulp of postpartum individuals, we applied shotgun proteomics to dental pulp tissues obtained from postpartum females.
- Our results confirmed the compositional width of the dental pulp proteome, which is a useful reference for interpreting dental pulp proteome results obtained in the context of forensic and archaeological science.
- Although pregnancy‐specific protein was not identified, this study provides important contributions to narrowing down the approaches that should be adopted to develop novel protein biomarkers in future research.

Because the DNA of blood‐borne pathogens has been identified in dental pulp, it is expected that blood circulating proteins that reflect the physiological status of the host individual can also be detected in the dental pulp. Despite the widespread use of DNA, proteins entrapped in dental pulp have attracted little attention. Proteins are the functional agents of body tissues and provide biological information, such as the physiological status of the host, interaction between the host and pathogens, host sex, and postmortem interval, which sometimes cannot be determined from DNA [9, 10, 11, 12, 13, 14, 15]. Mass spectrometry‐based proteomics has been applied to comprehensively identify the proteins present using liquid chromatography–tandem mass spectrometry (LC–MS/MS), where various numbers of proteins, from 66 to 4332, have been identified in healthy human dental pulp samples [16, 17, 18, 19, 20, 21]. Presumable peptides originating from the bacterial proteins of *Yersinia pestis* were identified in the dental pulp of human skeletons that were excavated from a documented plague site in France ∼300 years ago [22]. In addition, the presence of *Staphylococcus aureus* was suggested by proteomic analysis of dental pulp from a human skeleton in Italy, which may possibly be the famous painter Caravaggio (1571–1610) [23]. Mass spectrometry‐based peptide mass fingerprinting of dental pulp is used to taxonomically classify ancient mammals [24].

This study explores the potential use of pulp proteomics for the forensic identification of individuals who are pregnant or postpartum. Specifically, we investigated the protein composition of human dental pulp samples from individuals with a known history of pregnancy and delivery. Our objective was to assess the possibility of determining whether the individuals were in their gestational period or postpartum phase at the time of death based on the identification of physiologically informative specific proteins present in their dental pulp. If an unidentified body is found to be pregnant or shortly after delivery, this may facilitate individual identification. If the child can be identified, then the identity of the woman can be determined through a maternity test.

Several unique proteins are expressed in the placenta during pregnancy, and some of these pregnancy‐specific proteins circulate in the maternal body tissues via the bloodstream [25] and possibly remain present for several weeks or months after delivery. For example, human chorionic gonadotropin (hCG) is a peptide hormone that affects various aspects of human pregnancy, such as facilitating the production of progesterone and suppressing the maternal immunological response. Enzyme immune assay revealed that serum concentrations of hCG rapidly increase after implantation, peak around 8 weeks post‐implantation [26], and decrease 2–3 weeks before delivery [27]; however, it remains detectable for a few weeks after delivery [28]. Alpha‐fetoprotein (AFP) is a major plasma protein expressed in the yolk sac and fetal liver. AFP functions in a manner that is similar to that of serum albumin in adults, and its concentration is the highest just after delivery (14080 ± 5944 µg/mL) and decreases during the first 2 weeks after birth (70.21 ± 52.92 µg/mL) in dogs [29]. Because these proteins circulate in the bloodstream, it is probable that they reach the pulp chambers and are entrapped in the dental pulp. The abundance of these pregnancy‐specific proteins in dental pulp would be low; therefore, they may not form a clear band in gel electrophoresis. However, we can expect that these proteins will be identified by shotgun proteomics, which comprehensively analyzes proteins with greater sensitivity.

## 2 Materials and Methods

### 2.1 Sample Collection

A total of eight dental pulp samples were collected from patients who wished to extract their third molars during routine dental treatment at the University of Yamanashi Hospital. Five samples were obtained from four female patients who had recently given birth (4–12 months postpartum), and three samples were obtained from nulliparous female or male patients (Table 1). For convenience, in this study, the former is referred to as postpartum samples, and the latter is referred to as control samples. Since females may have experienced conception and early miscarriage without being aware of it, one male sample was included as a definitive negative control. There is one case in which two samples were obtained from the same individual. This was done to examine changes in the detectability of pregnancy‐specific proteins over time postpartum. No teeth were affected by caries. Only teeth extracted for treatment were included, and no teeth were extracted solely for use in this study. Sample collection was performed with the written informed consent of the patients. The research design was reviewed and approved by the ethics committee of the University of Yamanashi Hospital (Approval number: 1781). Also, this study was conducted in accordance with the Code of Ethics of the World Medical Association (Declaration of Helsinki).

**TABLE 1:** Information on the dental pulp samples used in this study.

ID	Sex	Age	Classification	Postpartum (days)	Element	Amount (mg)	Note
UYH01	F	25	Postpartum	126	Upper right	2.7	Same individual with UYH02
UYH02	F	25	Postpartum	157	Upper left	1.8	Same individual with UYH01
UYH04	F	31	Postpartum	175	Upper left	2.1	
UYH07	F	34	Postpartum	269	Upper right	N.A.	
UYH10	F	43	Postpartum	354	Upper right	1.6	
UYH15	F	43	Control	—	Upper right	2.1	
UYH16	F	27	Control	—	Upper right	N.A.	
UYH19	M	35	Control	—	Upper left	N.A.	

The third molars were first freeze‐dried and then cut with a dental drill and diamond disk at the horizontal plane of the enamel–cementum junction. The dental pulp was extracted with stainless steel tweezers and placed inside a Protein LoBind tube. The dental drill, diamond disk, and tweezers were thoroughly cleaned with 100% ethanol under ultrasonication to prevent cross‐contamination.

### 2.2 Proteomic Analysis

The protein extraction protocol followed the previously published method by Eckhardt et al. [17]. Approximately 2 mg of dried dental pulp sample was suspended in 300 µL of lysis buffer (7 M urea, 2 M thiourea, and 50 mM NH_4_HCO_3_) and disrupted via ultrasonication at 4°C for 15 min. Samples were centrifuged, and the supernatants were collected in new Protein LoBind tubes. The supernatant fractions were concentrated using molecular cutoff filters (Amicon Ultra 3K) and resuspended in 50 mM ammonium bicarbonate buffer. The protein concentration was measured using the Bradford assay, and 10 µg of protein was separated for trypsin digestion. The samples were reduced with 10 mM DTT, alkylated with 25 mM IAA, and digested with 0.5 µg trypsin at 37°C for 16 h. The digested peptides were desalted by purification using a reverse phase C18 tip column (StageTips) [30], and the solution was evaporated with a SpeedVac. A laboratory blank that does not contain sample was processed with the same manner.

Dried peptides were resuspended in 0.1% formic acid and 2% ACN, and 1.0 µg peptides were analyzed using an Orbitrap Elite; Hybrid Ion Trap‐Orbitrap Mass spectrometer (Thermo Fisher Scientific) coupled with an UltiMate 3000 HPLC system (Thermo Fisher Scientific). The analytical conditions were as follows: Trap column (Acclaim PepMap 100; 100 µm × 20 mm, C18, 5 µm, 100 Å), Nano HPLC capillary column (75 µm × 125 mm, C18, 3 µm; Nikkyo Technos Co., Ltd., Tokyo, Japan); mobile phase = (A) 0.1% formic acid and 2% ACN, (B) 0.1% formic acid and 95% ACN; gradient, A:B = 98:2 (0 min), 67:33 (120 min), 10:90 (120–130 min); flow rate = 300 nL/min; mass scan range = *m/z* 380–1500; mass resolution = 60,000; charge state screening = enable; unassigned charge states = 2+ and 3+ were not rejected; normalized collision energy = 35.0 eV.

The resultant raw files were searched against a human proteome (UP000005640) obtained from Uniprot (downloaded on March 10, 2024) with MaxQuant software [31], version 2.2.0.0. The parameters used for the analysis were those preset for Orbitraps for parent mass error and fragment mass tolerance. Carbamidomethylation of cysteine was set as a fixed modification, and acetylation of the N‐terminus, deamidation of asparagine and glutamine, and the derivation of pyroglutamic acid were set as variable modifications. A maximum of five modifications per peptide were allowed, and all peptides were automatically filtered by a false discovery rate of 1.0% and manually filtered by at least two different non‐overlapping peptides. Protein groups with scores below 4.3 were excluded from the results. Since the score is defined as the ‐10 logarithmic probability of observing the given number of matches or more by chance [31], this roughly corresponds to the probability of 5e^−5^. All contaminant accessions (i.e., keratins and trypsin) were excluded from further analysis using the contamination.fasta provided by MaxQuant, which includes common laboratory contaminants. Proteins identified from the blank were also excluded from further analysis.

### 2.3 Data Analyses

The gene ontology (GO) of the identified proteins was compared using the PANTHER database, version 18.0 [32]. The R software environment, version 4.2.3, was used to process, analyze, and visualize the data [33]. Principal component analysis (PCA) was applied to the spectral intensities of each protein group, with zero replacement by one and log transformation. The upset plot was drawn by using UpSetR package in R [34].

Dental pulp proteomes reported in previous studies were compiled to compare with the results obtained in this study. If mass spectrometry. raw data were uploaded to a public repository, they were analyzed in the same workflow as this study [17]. If only tables were shown and. raw data are not available, the leading razer proteins were used for the comparison after excluding contaminants and proteins with only one unique peptide assigned [18, 19, 20]. Data reported by Silva et al. [21] were not used due to the lack of a comprehensive list of identified proteins from healthy dental pulp samples. Data reported by Eckhard et al. [16] were also not used because a labor‐intensive but thorough protein identification method is not readily applicable to routine forensic or archaeological investigations.

## 3 Results

### 3.1 Overall Profiles

**FIGURE 1:**
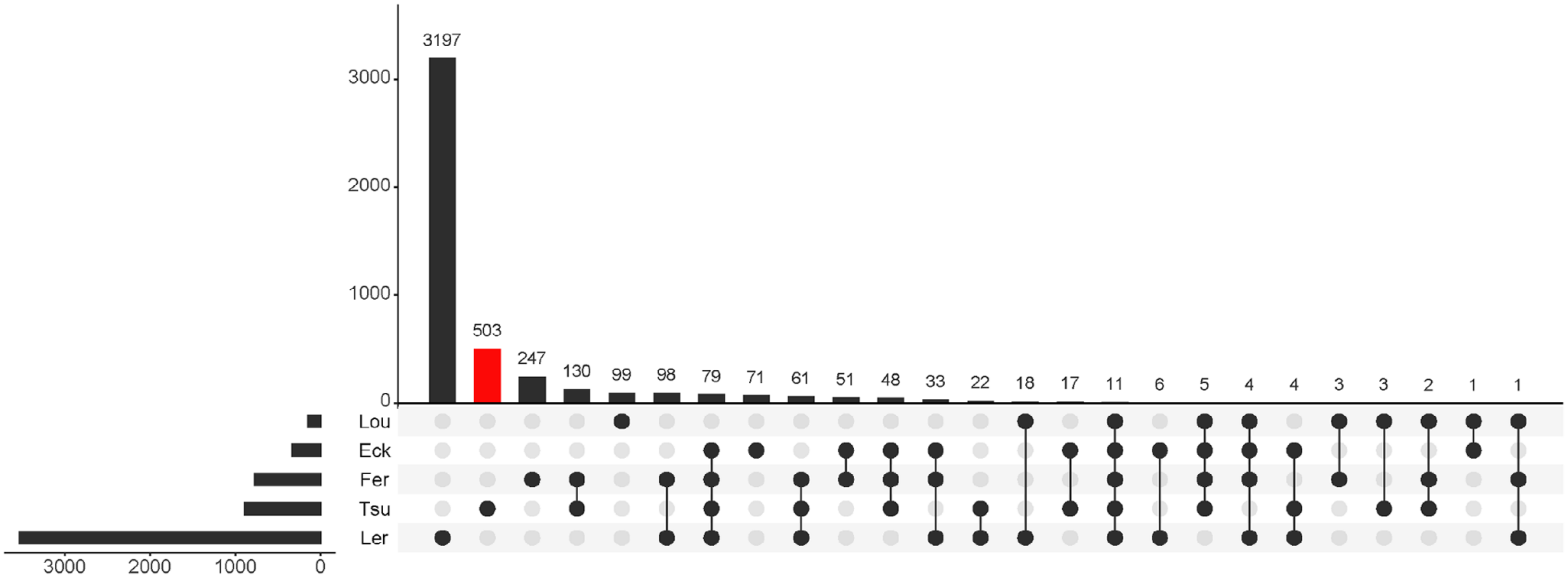
Upset plot showing the intersection of the identified proteins in proteomic studies of human dental pulp. Lou, Loureiro et al. [20]; Eck, Eckhardt et al. [17]; Fer, Feridouni Khamaneh et al. [18]; Ler, Tsu, this study; Lertruangpanya et al. [19]. The subgroup of proteins identified only in this study is colored red. Note that the proteomic data analysis was not carried out with the same workflow, the comparison is just a reference.

**FIGURE 2:**
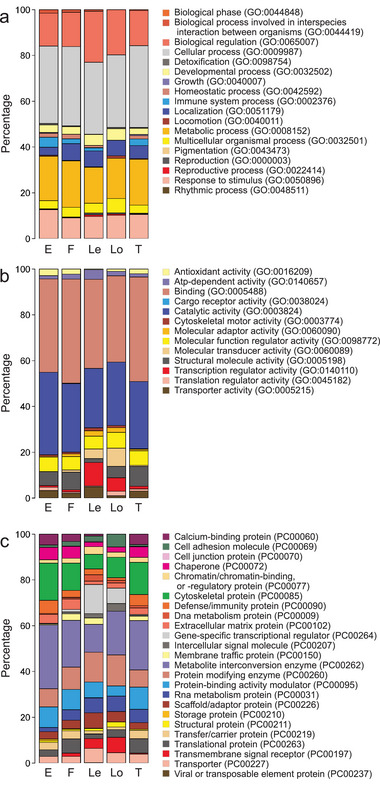
Comparison of the PANTHER gene ontology protein classification results in the (a) biological process, (b) molecular function, and (c) protein class between Eckhardt et al. [17], “E”; Feridouni Khamaneh et al. [18], “F”; Lertruangpanya et al. [19], “Le”; Loureiro et al. [20], “Lo”; and this study, “T”.

A total of 885 protein groups that had ≥ 2 unique + razor peptides were identified from the eight dental pulp samples, after excluding low‐score or possible contaminant proteins (Table S1). Although there was relatively little overlap of identified proteins among different studies (Figure 1), the composition of the PANTHER GO classifications in the biological process, molecular function, and protein class was similar (Figure 2).

### 3.2 Pregnancy‐Specific Proteins

Contrary to expectations, pregnancy‐specific proteins that are only expressed during pregnancy and delivery were not included in the 885 identified proteins. There were nine proteins that were only identified in the postpartum samples (4–12 months postpartum) and not in the control samples (Table 2). Although it is possible that these proteins may be related to pregnancy, no protein is reported to be exclusively expressed during pregnancy.

**TABLE 2:** Detailed results of the nine proteins that were only identified in postpartum samples (4–12 months postpartum) but not in the control samples.

Uniprot ID	Gene name	Protein name	Number of unique+razor peptides	Unique+razor sequence coverage (%)	Score
P10915	HAPLN1	Hyaluronan and proteoglycan link protein 1	5	24	15.218
O15212	PFDN6	Prefoldin subunit 6	4	45.7	35.422
Q5VWC4	PSMD4	Reticulocalbin‐2	3	21.8	5.1916
Q14257	RCN2	26S proteasome non‐ATPase regulatory subunit 4	3	8.7	9.0469
P09497	CLTB	Isoform 2 of Cytochrome b5	2	39.8	12.594
Q96FJ2	DYNLL2	Clathrin light chain B	2	10	19.83
P23141‐3	CES1	Isoform 3 of Liver carboxylesterase 1	2	6.5	7.4933
P00167‐2	CYB5A	Dynein light chain 2, cytoplasmic	2	39.3	4.6001
Q96HN2‐2	AHCYL2	Isoform 2 of Adenosylhomocysteinase 3	2	5.6	4.478

### 3.3 Principal Component Analysis

**FIGURE 3:**
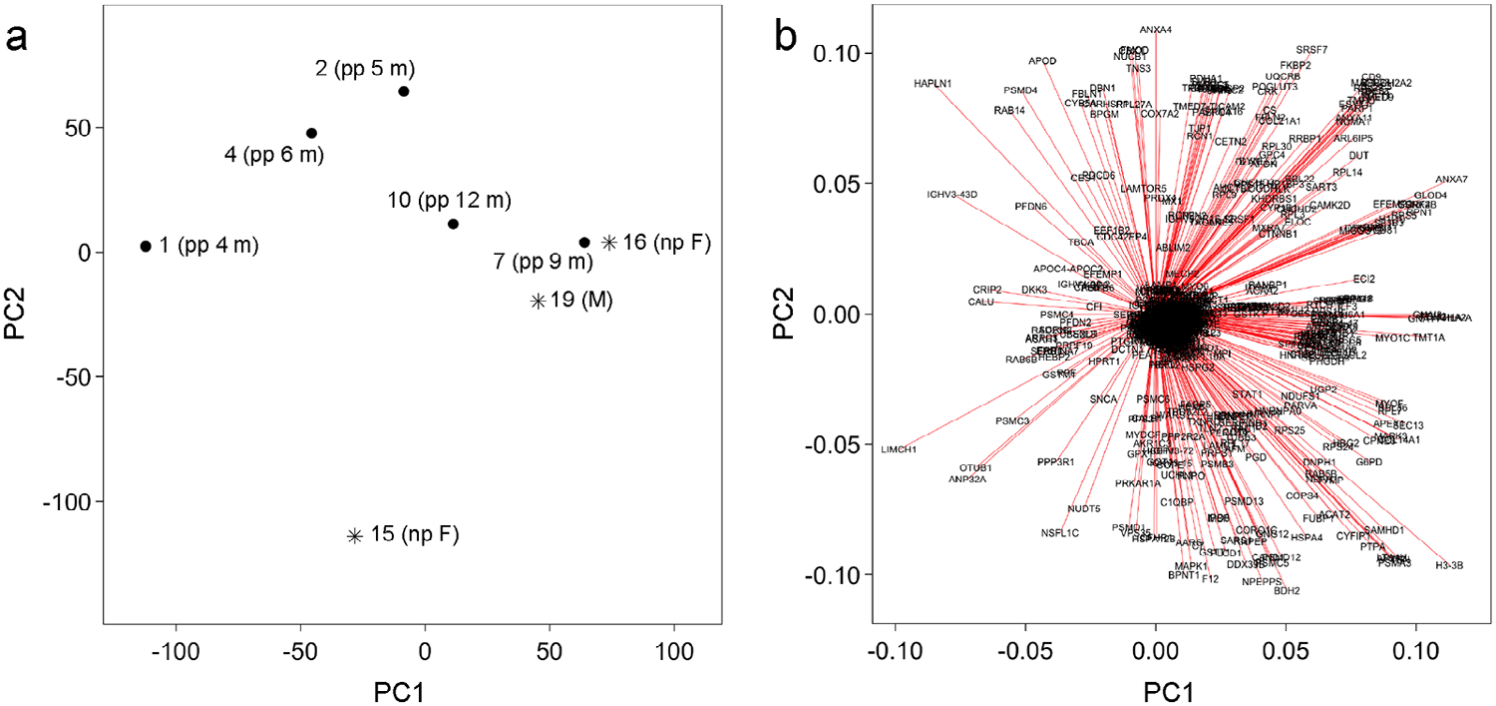
Results of the (a) principal component analysis (PCA) of each individual and (b) loading of each protein group. The sample name and subject individual identifiers (pp, postpartum; np, nulliparous; F, female; and M, male) are presented in the PCA plot.

The dental pulp proteomes obtained from the three individuals who were postpartum for 4, 5, and 6 months appeared to be separate from those of other individuals (9 and 12 months postpartum and control samples) by PCA (Figure 3a). However, PC1 and PC2 only explained 24.9% and 18.2% of the variance, respectively. Furthermore, there were no proteins that exhibited a distinct degree of loading (Figure 3b), and it was unclear which protein or group of proteins induced these differences. Therefore, conclusive evidence was not obtained from PCA.

## 4 Discussion

Shotgun proteomics employed in this study expanded the dental pulp proteome with the addition of approximately 500 proteins at the maximum (Figure 1). In studies where the. raw data from mass spectrometry is not available, the data analysis could not be carried out with the same workflow as this study. Therefore, the comparison is not rigorous, and the results for the intersection of protein lists obtained from different studies should be regarded as a reference (Figure 1). Although it is possible that tiny amounts of enamel, dentin, and cementum powder were contaminated in the dental pulp during the pulp extraction procedure, their contribution would be minimal because decalcification, an essential step to extract proteins from calcified hard tissues, was not performed in this study. A comprehensive list of proteins present in dental pulp is useful to interpret present pathological conditions. For example, if nontypical immunological or defense proteins are identified in dental pulp, it can be interpreted that the host individual experienced severe stresses, such as disease, infection, and inflammation [20]. Furthermore, a comprehensive pulp protein list is useful to assess the authenticity of the endogenous origin of proteins retrieved from degraded dental pulp in the context of forensic and archaeological sciences. For example, if the proteome identified from a degraded dental pulp sample contains a large population of previously unidentified proteins, it is likely contaminated by exogenous proteins. The identification of hundreds to thousands of various proteins from dental pulp supports the clinical evidence that dental pulp is a dynamic tissue that functions in several aspects, such as protection from bacterial invasion, dentin regeneration throughout life, immune defense reactions, and sensory recognition [5].

The results of this study suggest that shotgun proteomics of bulk dental pulp was not a useful method to discriminate whether the host was pregnant or recently postpartum, at least for individuals that were 4 months postpartum or later. This result is in line with a previous proteomic study on ancient dental pulp that sometimes failed to detect bacterial proteins originating from *Y. pestis* even in plague‐positive individuals who were diagnosed by ancient DNA analysis [22]. However, two major issues hindered the detection of pregnancy‐specific proteins in this study. First, the dental pulp samples were obtained from individuals who were 4 months postpartum or later in this study, and no individuals who were currently pregnant or a few days or weeks postpartum were included. Available evidence showed that the maternal serum concentrations of some pregnancy‐specific proteins, such as hCG and AFP, rapidly decreased before or after a few weeks postpartum [27, 29]. Although this is not necessarily applicable to other pregnancy‐specific proteins, it is possible that the timeframe for the detection of pregnancy‐specific proteins may be narrow, especially after delivery. Second, it is possible that low‐abundance pregnancy‐specific proteins were present, but they were masked by other abundant proteins and, therefore, not identified by mass spectrometry analysis. A large portion of peptides are not sequenced in data‐dependent acquisition, which is the common strategy for the routine application of proteomics in life science because of the limited acquisition capacity and speed [35]. Because of these two issues, it is possible that pregnancy‐specific proteins are present in dental pulp, but the study design was not suitable to detect them.

Although the dental pulp proteomes that were obtained from individuals ≤6 months postpartum appeared to form a different cluster compared with individuals ≥9 postpartum and control individuals (Figure 3a), their driving cause was less evident. PC1 and PC2 together only explained 43.1% of the variance, and no protein exhibited distinct loading (Figure 3b). Because pregnancy induces changes in protein metabolism throughout the body [36], these effects may persist postpartum and are detected in the dental pulp. However, it is necessary to investigate whether the same pattern of dental proteome remains when the number of individuals is expanded to confirm this possibility. Nevertheless, the driving factor for the apparent difference observed in PCA remains unclear.

Sample collection, protein extraction, mass spectrometry analysis, and research design could be improved in future studies to detect pregnancy‐specific proteins in dental pulp for forensic applications. First, it is necessary to obtain dental pulp samples from subjects who definitely have circulating pregnancy‐specific proteins in their blood as a positive control. However, dental treatment for individuals who are pregnant requires special care [37, 38], and tooth extraction is usually very rare during pregnancy; therefore, obtaining such intact tooth samples is challenging. Experimental studies using non‐human animals may be an alternative approach. However, the morphology of the high‐crowned teeth of herbivores differs from that of human teeth, which have closed crowns, and direct comparisons cannot be made [39]. In addition, direct comparisons between humans and non‐human animals may not be possible, as they do not necessarily have similar gestation periods and physiological conditions during pregnancy. Second, protein extraction methods can be improved. Blood circulating proteins are expected to be trapped more loosely in the pulp chamber than the structural proteins that form the bulk of dental pulp. Therefore, it would be possible to increase the relative concentration of the secreted proteins while mostly eliminating the structural proteins that originate from the extracellular matrix by stopping protein extraction at very short periods. This approach of short‐time extraction has been shown to be effective in the detection of pathogens by ancient DNA analysis [40]. Third, the targeted approach would allow for more sensitive detection. The retention time and mass range of pregnancy‐specific proteins identified in the blood of women who are pregnant [25] could be used to identify the same pregnancy‐specific proteins with increased sensitivity in dental pulp by adopting multiple reaction monitoring [41, 42]. Alternatively, enzyme‐linked immunosorbent assay (ELISA) can be used for protein marker identifications as it is a more sensitive method [43], although the applicability of ELISA to archaeological materials is debated [44]. Finally, a basic understanding of the behaviors and dynamics of pregnancy‐specific proteins is necessary to clarify whether pregnancy‐specific proteins circulate in the maternal bloodstream but are difficult to detect in dental pulp or whether they are totally absent from the bloodstream, at a given postpartum age. Research on the distribution and dynamics of pregnancy‐specific proteins within the body is surprisingly scarce, likely because they are not directly related to the early diagnosis or treatment of diseases. Future research should first establish basic data by analyzing maternal blood to determine which pregnancy‐specific proteins persist postpartum, for how long, and at what concentrations. Such basic data would enable the development of targeted, highly sensitive detection approaches as well.

In conclusion, contrary to our expectations, pregnancy‐specific proteins were not identified in human dental pulp obtained from individuals 4–12 months postpartum. This may be because pregnancy‐specific proteins were removed from the maternal body by 4 months postpartum, or the abundance of pregnancy‐specific proteins was so low that they were masked by other more abundant proteins in the mass spectrometry analysis. However, this study provides important contributions to narrowing down the approaches that should be adopted to develop novel protein biomarkers in future research for forensic science. Compared with DNA, proteins provide information on the host's physiological condition, and retrieving this information can improve forensic and archaeological identification of dead individuals.

## Author Contributions

Conceptualization: Takumi Tsutaya and Noboru Adachi. Methodology: Takumi Tsutaya, Kana Fujimoto, Yusuke Nakai, Koichiro Ueki, Yayoi Kimura and Noboru Adachi. Software: Takumi Tsutaya. Investigation: Takumi Tsutaya, Yusuke Nakai, and Yayoi Kimura. Resources: Kana Fujimoto, Naana Mori, Ran Iguchi, Akinori Moroi, Kunio Yoshizawa, Koichiro Ueki, and Noboru Adachi. Writing–original draft: Takumi Tsutaya, Yayoi Kimura, and Noboru Adachi. Visualization: Takumi Tsutaya. Funding acquisition: Takumi Tsutaya and Yayoi Kimura.

## Conflicts of Interest

The authors declare no conflicts of interest.

## Supporting information



Supporting Information

## Data Availability

The mass spectrometry results were uploaded to the PRIDE repository with the identifier PXD052602 [45].
